# Developing Bi-Gold Compound BGC2a to Target Mitochondria for the Elimination of Cancer Cells

**DOI:** 10.3390/ijms232012169

**Published:** 2022-10-12

**Authors:** Qingbin Cui, Wenwen Ding, Panpan Liu, Bingling Luo, Jing Yang, Wenhua Lu, Yumin Hu, Peng Huang, Shijun Wen

**Affiliations:** State Key Laboratory of Oncology in South China, Collaborative Innovation Center for Cancer Medicine, Department of Experimental Research, Sun Yat-sen University Cancer Center, Guangzhou 510060, China

**Keywords:** gold complex, anticancer, TrxR, mitochondria, energy metabolism

## Abstract

Reactive oxygen species (ROS) homeostasis and mitochondrial metabolism are critical for the survival of cancer cells, including cancer stem cells (CSCs), which often cause drug resistance and cancer relapse. Auranofin is a mono-gold anti-rheumatic drug, and it has been repurposed as an anticancer agent working by the induction of both ROS increase and mitochondrial dysfunction. Hypothetically, increasing auranofin’s positive charges via incorporating more gold atoms to enhance its mitochondria-targeting capacity could enhance its anti-cancer efficacy. Hence, in this work, both mono-gold and bi-gold compounds were designed and evaluated to test our hypothesis. The results showed that bi-gold compounds generally suppressed cancer cells proliferation better than their mono-gold counterparts. The most potent compound, BGC2a, substantially inhibited the antioxidant enzyme TrxR and increased the cellular ROS. BGC2a induced cell apoptosis, which could not be reversed by the antioxidant agent vitamin C, implying that the ROS induced by TrxR inhibition might not be the decisive cause of cell death. As expected, a significant proportion of BGC2a accumulated within mitochondria, likely contributing to mitochondrial dysfunction, which was further confirmed by measuring oxygen consumption rate, mitochondrial membrane potential, and ATP production. Moreover, BGC2a inhibited colony formation and reduced stem-like side population (SP) cells of A549. Finally, the compound effectively suppressed the tumor growth of both A549 and PANC-1 xenografts. Our study showed that mitochondrial disturbance may be gold-based compounds’ major lethal factor in eradicating cancer cells, providing a new approach to developing potent gold-based anti-cancer drugs by increasing mitochondria-targeting capacity.

## 1. Introduction

**Figure 1:**
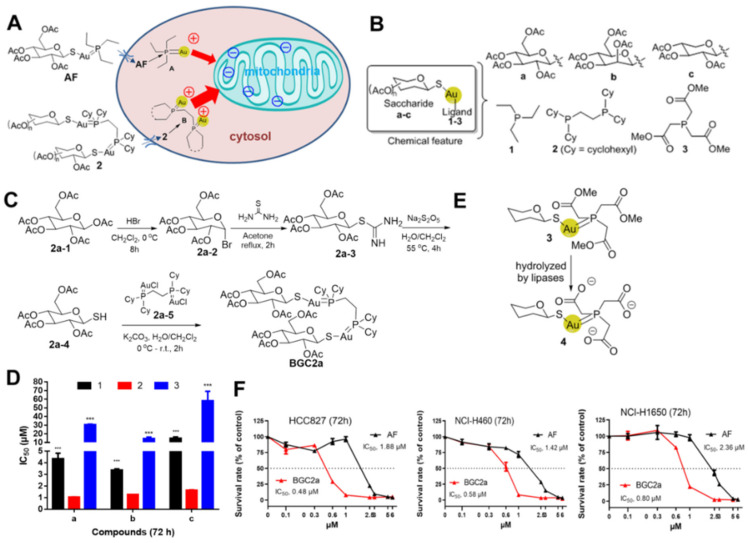
Auranofin (AF), the designed bi-gold compound BGC2a and their antiproliferation effects on lung cancer cells. (**A**) Our hypothesized action model: targeting mitochondria by gold compounds with different degrees of positive charges. (**B**) The designed gold compounds with various saccharide thiols (a–c) and phosphine ligands (1–3). (**C**) Synthetic route of BGC2a. Note: Cy means cyclohexyl. (**D**) IC_50_ values of different gold-containing compounds against A549 lung cancer cells after a 72 h treatment. Series 2 bi-gold compounds exhibited much lower IC_50_ values compared to both series 1 compounds and series 3 compounds. IC_50_ values were calculated by GraphPad Prism 7 for their half response to inhibit cell proliferation. (***) *p* < 0.001 vs. series 2. (**E**) The proposed intracellular hydrolysis of compound 3 to become negative-charged compound 4. (**F**) The antiproliferation activities of BGC2a and AF in three other lung cancer cell lines HCC827, NCI,-H460 and NCI-H1650.

Medicines formulated with gold (Au)-containing compounds have been employed to treat certain diseases for centuries [1,2]. For example, Auranofin (AF), a typical mono-gold compound, was approved for the treatment of rheumatic arthritis. Additionally, AF has been reported to possess inhibitory effects towards a broad spectrum of cancer cells, including Hodgkin lymphoma, colon cancer, and lung cancer cells [3,4,5]. It has been conceptualized that ^+^Au-PEt_3_ (Figure 1A), the active pharmacophore of AF, can bind to the active site of an antioxidant enzyme thioredoxin reductase (TrxR) through the formation of Au-S or Au-Se bonds [6]. TrxR is often upregulated in cancer cells for maintaining cellular redox balance [7,8]. Its inhibition usually results in an increased level of reactive oxygen species (ROS), which can then damage cell components, leading to DNA damage and apoptosis [9,10,11]. While AF is now under multiple clinical trials for cancer therapy by single administration or combination with other conventional chemotherapies (NCT03456700, NCT01737502), any breakthrough progress remains to be released. Thus, novel gold-based compounds with highly potent anti-cancer activity are still in high demand.

Altered energy metabolism is one of the hallmarks of cancers [12]. It is known that cancer cells tend to down-regulate oxidative phosphorylation (OXPHOS) and switch to anaerobic ATP generation to repurpose mitochondrial metabolism for the synthesis of building blocks for cancer cell proliferation, including nucleotide precursor synthesis (a term called Warburg effect that serves as a therapeutic target in cancer) [13]. However, mitochondria in cancer cells still play key roles in cell proliferation for their energy supply and apoptotic events initiation. Both energy supply and apoptotic events can be targeted and regulated by small-molecule drugs to induce cell death via ATP exhaustion and apoptotic induction [14]. Compared with normal cells, mitochondria in cancer cells exhibit functional and structural alterations, and this alteration renders cancer cells more sensitive to further disturbance, which serves as a biochemical basis for targeting mitochondria for cancer treatment [15]. Some literature has demonstrated that AF could disturb mitochondrial respiration to suppress cancer growth [16,17], implying that gold compounds with more capacity to target mitochondria may possess higher anti-cancer activity. Generally, mitochondrial inner membranes are negatively charged and can be preferentially attacked by those positively charged molecules [15,18], e.g., the active form of AF, Au^+^-PEt_3_. Theoretically, gold compounds with a bi-gold scaffold might have higher anti-cancer potential. Compared to mono-gold compounds bearing only one Au^+^, these compounds with two Au^+^ may be superior to targeting the negatively charged mitochondria, as illustrated in Figure 1A [6,19]. Herein, we report a novel bi-gold compound 2a (BGC2a) that substantially inhibited cancer cell proliferation in vitro and prevented tumor growth in vivo. Mechanistically, BGC2a accumulated in mitochondria, intervened mitochondrial function, substantially impacted cancer cell stemness, and inhibited colony formation. We further confirmed that disturbing mitochondrial function might be the major lethal factor, although the inhibition of TrxR could not be excluded.

## 2. Results

### 2.1. Bi-Gold Compounds Showed Better Anti-Cancer Capacity In Vitro

To test our hypothesis that the increase of positive charge could generally gain higher anticancer potency, we designed and synthesized three series of gold-containing compounds. These compounds contained either one Au atom (series 1 and 3) or two Au atoms (series 2) by varying phosphine ligands (monophosphine for series 1, biphosphine for series 2, and monophosphine with triester bonds attached for series 3) or thiocarbohydrates (glucose for a, mannose for b, and arabinose for c) as shown in Figure 1B. The synthesis of BGC2a, as an example, began with acetated thioglucose 2a-4 reacting with freshly prepared AuCl-phosphine complexes 2a-5 using potassium carbonate as a base in water/dichloromethane (Figure 1C, see details in the Supplementary Materials).

After the synthesis and structural characterization of the aforementioned gold compounds, their antiproliferative effects on lung cancer cell line A549 were first examined. The results showed that series 1 compounds, including AF (labeled as 1a) bearing single-ion core (Au^+^-PEt_3_) exhibited moderate IC_50_ values (Figure 1D). On the other hand, series 2 compounds, including BGC2a (labeled as 2a) with a double-gold-ions core, exhibited much lower IC_50_ values, with potency 3− to 6−fold higher than their counterpart mono-gold series 1 compounds. By contrast, ester-chelated mono-gold series 3 compounds showed very weak antiproliferative activity, likely due to their incapability to enter mitochondria after intracellular hydrolysis to generate negatively charged carboxylic acids 4 (Figure 1E). In addition, the pure corresponding tricarboxylic acids 4 of series 3 compounds did not show anti-cancer activity at 100 μM (data not shown). Taken together, these results suggested that the better ability of targeting mitochondria by BGC2a might cause its cytotoxicity to be higher than AF. In addition, we tested the cytotoxicity of BGC2a in Kras inducible HEK-293 cells that can mimic cancer cell phenotype when Kras is on [20]. The results showed that Kras on cells were more sensitive to BGC2a than Kras off cells (Figure S1), indicating that BGC2a might potentially target cancer cells over normal cells, although further study is still needed to verify.

BGC2a was finally selected to be further evaluated in the other three lung cancer cell lines, HCC827, NCI-H460, and NCI-H1650. The results shown in Figure 1F confirmed that BGC2a was consistently more potent than AF. Meanwhile, BGC2a was tested in other types of cancer cell lines, including pancreatic (CAPAN-2 and PANC-1), melanoma (B16), and murine acute myeloid leukemia (BaF3-ITD). Compared to AF, BGC2a more effectively inhibited the cells proliferation in a dose-dependent manner (Figure S2). To investigate the effect of the hydrophobic cyclohexyl of BGC2a on the gold ion delivery, a mono-gold compound MGC2a that has tricyclohexylphosphine only to replace triethylphosphine in AF was synthesized. The antiproliferative activity of MGC2a was much less potent than BGC2a (Figure S3), again validating our hypothesis that the increased positive charge indeed contributed to the anti-cancer potency of bi-gold compounds.

### 2.2. BGC2a Inhibited Cellular TrxR, Induced Apoptosis, and Increased ROS

**Figure 2:**
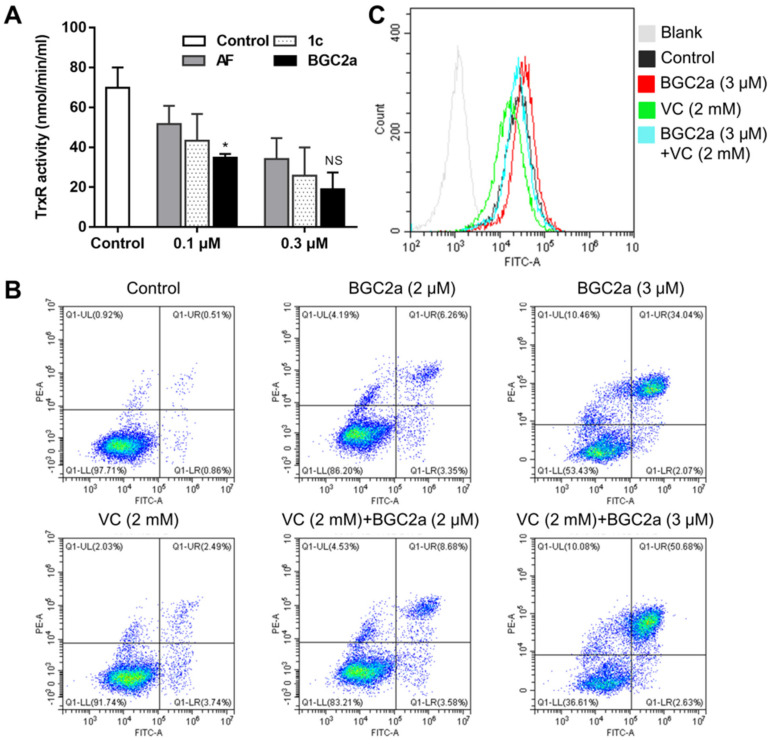
The effects of AF and BGC2a on cellular TrxR, ROS, and apoptosis induction in A549 cells. (**A**) After treatment by AF and BGC2a at 0.1 and 0.3 μM for 2 h, cellular TrxR enzymatic activity was measured by TrxR Activity Colorimetric Assay Kit. (*) *p* < 0.05. (**B**) The induced apoptosis after 24 h treatment by AF and BGC2a at 2 and 3 μM in A549 cells. Pretreatment with VC at 3 mM was applied to study a reversal effect on BGC2a-induced apoptosis. (**C**) The increased global ROS after 6 h treatment by AF and BGC2a at 3 μM in A549 cells. CM−H_2_DCFDA (6 μM, 30 min) and flow cytometry were applied to test the global ROS production. Pretreatment with VC at 3 mM for 2 h was applied to study the reversal effect on BGC2a-induced ROS.

Since the series 2 bi-gold compounds, including BGC2a, were verified to be more potent than AF in inhibiting cell proliferation, we next determined their effects on cellular TrxR, a typically reported target of AF and other gold-based compounds [21]. As shown in Figure 2A, after 4 h treatment, BGC2a exhibited dose-dependent inhibitory effects against cellular TrxR, slightly better than AF. Meanwhile, BGC2a at 3 μM significantly induced apoptosis in A549 cells (Figure 2B). Compared to BGC2a, AF only caused a moderate apoptotic effect at a higher dose (5 μM as shown in Figure S4). Due to the TrxR inhibition, BGC2a also increased the cellular ROS levels (Figure 2C). To investigate the role of ROS in the BGC2a-induced apoptosis, vitamin C (VC) was then used as an antioxidant agent to perform the rescue experiments. However, VC failed to reverse the cell apoptosis, yet the reagent successfully reduced the ROS induced by BGC2a (Figure 2B,C), implying that ROS might not be the major cause of cell apoptosis and that other lethal factors may involve cell death, consistent with Arner’s work that TrxR knockdown had only a slight effect on cell growth of A549 cells [22].

### 2.3. BGC2a Suppressed Mitochondrial Functions

**Figure 3:**
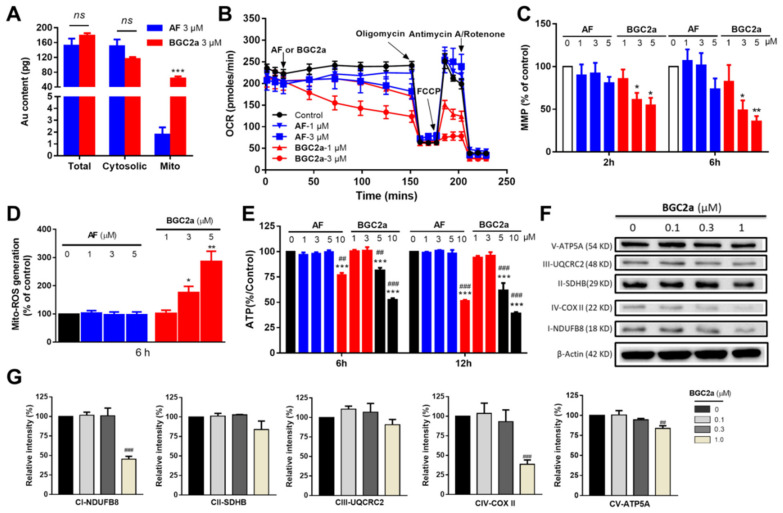
BGC2a accumulated in mitochondria and impacted mitochondrial functions of A549 cells. (**A**) The intracellular gold (Au) contents were detected after 2 h treatment with AF and BGC2a at 3 μM. Total intracellular and mitochondrial gold contents were measured by ICP-MS. (***) *p* < 0.001 vs. the AF group; Note: Mito stands for mitochondria. ns stands for no significant difference. (**B**) The effects on OCR by BGC2a and AF. Oxygen consumption was recorded by the Seahorse Bioscience Extracellular Flux Analyzer under basal conditions and upon oligomycin (1 μM), FCCP (1 μM), and Antimycin A/Rotenone (0.5 μM) at time courses. (**C**) The effect on MMP after 2 or 6 h treatment of AF and BGC2a. Rhodamine-123 (1 μM, 20 min) was used to stain the cells, and flow cytometry was used to detect the MMP. (*) *p* < 0.05, (**) *p* < 0.01 vs. the AF group. (**D**) Mitochondrial ROS production after 6 h treatment. MitoSOX™ Red (5 μM, 20 min) and flow cytometry were applied to test the ROS in mitochondria. (*) *p* < 0.05, (**) *p* < 0.01 vs. the AF group. (**E**) The ATP production after 6 h and 12 h treatment of BGC2a. (***) *p* < 0.001 vs. the AF group; (^##^) *p* < 0.01, and (^###^) *p* < 0.001 vs. the control group (one-way ANOVA). (**F**) The Western blot results of the electron transport chain complexes I-V (CI to CV) by measuring their core subunit protein levels and (**G**) their quantification after 24 h treatment of BGC2a. (^##^) *p* < 0.01, and (^###^) *p* < 0.001 vs. the control group. The resulting protein bands were quantified by using ImageJ software.

Mitochondria play key roles in the proliferation and growth of cancer cells for their energy supply and apoptotic event control [23,24,25]. As we hypothesized, the active form of gold compounds might facilitate the targeting of the negatively charged mitochondria, which might explain why the bi-gold compound BGC2a was more potent. To verify our hypothesis, the intracellular uptake and distribution of AF and BGC2a in whole cells and mitochondria were measured. After co-culturing with AF or BGC2a at 3 μM for 2 h in A549 cells, the whole cells and the isolated mitochondria were collected for gold detection using inductively coupled plasma-mass spectrometry (ICP-MS). As shown in Figure 3A, the total intracellular gold contents for both compounds were in a similar range, implying the uptake of BGC2a was not compromised, albeit with a bulky structure. However, a significant proportion of BGC2a accumulated in mitochondria, nearly 40-fold higher than AF (Figure 3A). Mechanistically, more positive charges of BGC2a due to the installed two Au atoms would facilitate its entrance in negatively charged mitochondria, thereby leading to the increased mitochondria-targeting ability. Finally, oxygen consumption rates (OCR), a key indicator of mitochondrial function, were measured, and the results showed that OCR was substantially inhibited by BGC2a (Figure 3B), more powerful than AF.

We next examined mitochondrial membrane potential (MMP) after treatments by AF and BGC2a (2 and 6 h) in A549 cells. Mitochondria demand high MMP not only to produce ATP continuously but also to maintain the basic cellular function to avoid apoptosis [26]. Therefore, decreasing MMP may lead to malfunctioning mitochondria and activated apoptosis [27]. Our results showed that BGC2a decreased MMP more efficiently than AF (Figure 3C). The substantial intervention of mitochondrial functions might disturb the electron transportation in the respiratory chain, which was subsequently verified by increased mitochondrial ROS induced by BGC2a (Figure 3D). It is worth mentioning that AF requires a higher concentration and a longer time to induce the same levels of ROS increase and MMP decrease (data not shown). Consequently, BGC2a suppressed the ATP production as measured by the CellTiter-Glo Luminescent Cell Viability Assay (Figure 3E). We noticed that the reduced level of ATP did not match the substantial inhibition of OCR, and one possible reason might be the glycolysis activation to compensate for the OCR intervention. Indeed, after examining the extracellular acidification rate (ECAR), we found that BGC2a (1, 3 μM) treatment increased the ECAR value, indicating the up-regulation of glycolysis (Figure S5).

Complexes I−V (CI−CV) in the respiratory chain are essential components in transporting electrons, serving as potential anti-cancer targets [28]. The Western blot results in Figure 3F,G indicated that BGC2a disrupted CI and CIV by measuring the protein levels of their core subunits, although the exact action mode remains to be further elucidated. Furthermore, our study showed that BGC2a treatment (1, 3, and 5 μM for 2h) did not alter the mitochondria mass (Figure S6).

### 2.4. BGC2a Inhibited Colony Formation, Reduced Side Population (SP) Cells, and Down-Regulated Cancer Stem Cell (CSC) Biomarkers

**Figure 4:**
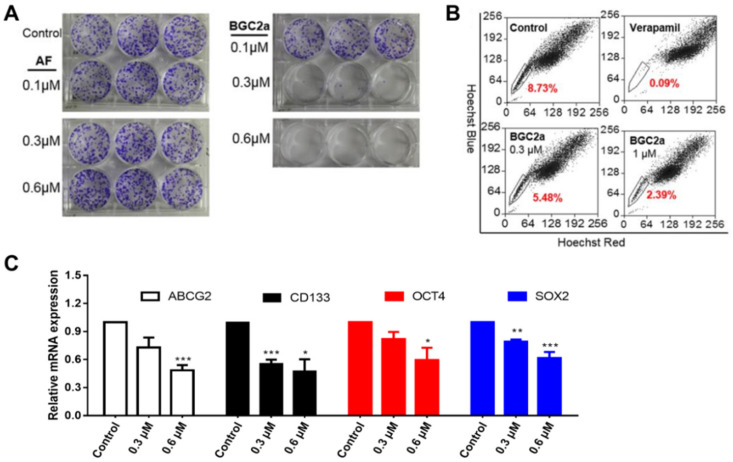
The effects of BGC2a in lung cancer cell CSCs. (**A**) The colony formation of A549 cells. 400 cells/well of A549 were treated for 14 days with AF or BGC2a at 0.1, 0.3, and 0.6 μM, respectively. (**B**) The SP cells of A549 cells were treated with BGC2a at 0.3 and 1 μM for 24 h using DMSO as the blank control and verapamil as the positive control. (**C**) CSC biomarkers of A549 cells indicated by RT-qPCR. A549 cells were treated with BGC2a at 0.3, 0.6 μM for 24 h to detect mRNA levels of ABCG2, CD133, OCT4, and SOX2. (*) *p* < 0.05, (**) *p* < 0.01, (***) *p* < 0.001 vs. the DMSO control group.

The effects of BGC2a on cancer stemness properties were further tested. The results showed that BGC2a at 0.3 μM could substantially reduce the colony number of A549 cells (Figure 4A). Since A549 cells reportedly exhibited a substantial amount of stem-like SP cells [29], we next tested the effect of BGC2a on SP. The data showed that BGC2a (0.3 and 1 μM) significantly reduced SP cells after 24 h treatment (Figure 4B). Finally, four well-established CSC biomarkers that play important roles in drug resistance [30] and tumorigenesis [31,32] were also tested: ATP-binding cassette subfamily G member 2 (ABCG2), prominin-1 (CD133), octamer-binding transcription factor 4 (OCT4), and sex-determining region Y-box 2 (SOX2). The treatment with BGC2a in A549 cells unambiguously decreased the mRNA levels of these four CSC biomarkers (Figure 4C).

### 2.5. BGC2a Suppressed Tumor Growth In Vivo

**Figure 5:**
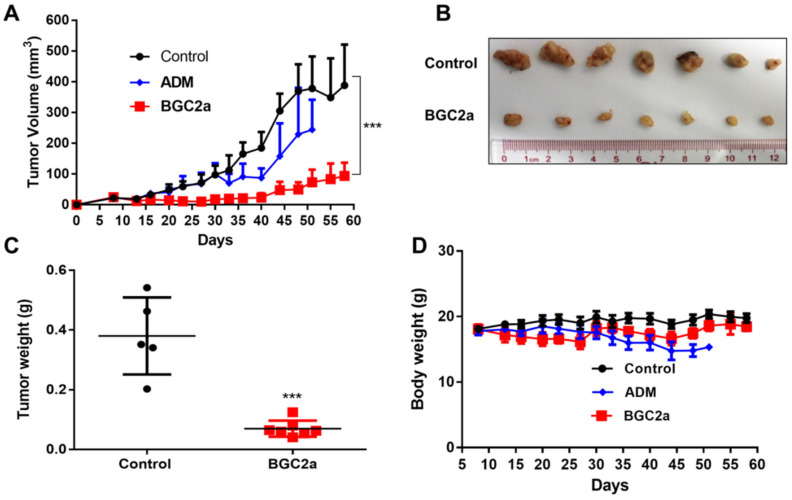
The inhibition of tumor growth in lung cancer A549 cells xenograft mouse model by BGC2a. (**A**) The measured tumor volumes. (***) *p* < 0.001 vs. the control group. (**B**) The tumors after the sacrifice of mice. (**C**) Quantification of the tumor weights from (**B**). (***) *p* < 0.001 vs. the control group. (**D**) Body weights of the treated mice. Mouse weight was measured 2 times a week.

To verify whether the in vitro anti-cancer effects could be translated to in vivo, A549 xenograft mice were treated with BGC2a at 3 mg/kg via intravenous injection 3 times a week. As shown in Figure 5A–C, after 50 days treatment, the tumor growth was significantly inhibited by BGC2a. The in vivo therapeutic effect of BGC2a appeared to be more effective than the positive control drug Adriamycin (ADM). More importantly, no significant body weight loss was observed at the end of the treatment of BGC2a, while the mice in the ADM-treated group lost more weight and died by day 50 (Figure 5D), indicating the relative safety of BGC2a. We also used PANC-1, a pancreatic cancer cell line with a highly malignant phenotype, as a second xenograft model for testing the anti-cancer activity in mice, and the results showed that tumor growth in the PANC-1 xenograft mice was significantly suppressed by BGC2a (Figure S7).

## 3. Discussion

Cancer cell proliferation, progression, and migration rely on a complex combination of altered cell cycle regulation [33], activated growth factor pathway [34], cell death resistance [35], and reprogrammed metabolism [36]. A better understanding of these mechanisms could lead to discovering potential targets and developing new strategies for the therapeutic intervention of tumor progression. Mitochondria are essential cellular organelles as the cell powerhouse [37]. Along with the production of ATP via the mitochondrial respiration chain is the efflux of protons, making the inner mitochondria negatively charged. Mitochondria in cancer cells are dysfunctional and more vulnerable to certain interventions than those in normal cells, providing a biochemical basis for mitochondria targeting as a potential therapeutic strategy against cancer [38]. Mitochondria also serve as a pivotal regulator of stemness in CSCs via modulating the proliferative potential, apoptosis, anabolism, and autophagy, which contribute to the progression and drug resistance of many tumors [39,40,41]. Energy metabolism aberration is one of the key hallmarks of CSCs [40]. CSCs in lung cancer, glioblastoma, and pancreatic cancer prefer the tricarboxylic acid cycle to generate ATP mediated by mitochondria [42,43,44], implying that targeting mitochondria could efficiently eradicate CSCs in these types of cancers.

AF was an approved drug for the treatment of rheumatic arthritis, and it was recently reported to have broad-spectrum anti-cancer effects mainly through the inhibition of the cellular antioxidant enzyme TrxR and the intervention of mitochondrial function [16,17]. In the current work, we confirmed that the anti-cancer effects of gold compounds, including AF and BGC2a, on malignant lung cancer cells and some other cancer cells. We hypothesized that targeting negatively charged mitochondria by the positive pharmacophore of AF might be the major factor in eliminating cancer cells. Therefore, increasing positive charges would enhance the ability to impact mitochondria, consequently gaining more potency. In our work, three series of gold compounds were designed and synthesized. The cell-based antiproliferative activity screening showed that all series 2 bi-gold compounds possessed relatively lower IC_50_ values. Bi-gold compound BGC2a proved to be the best, accumulating more in mitochondria than AF, which may contribute to BGC2a’s enhanced antiproliferative capacity. Moreover, to determine whether better shielding of positive charges by hydrophobic cyclohexyl groups of BGC2a might increase its mitochondrial accumulation, a mono-gold compound MGC2a with tricyclohexylphosphine ligand was synthesized and tested for its anti-cancer activity. Compared to BGC2a, MGC2a exhibited less potency in inhibiting the proliferation of lung cancer cells (Figure S3). These results verified that the increased positive charge, but not the shielding of positive charges, played a central role in enhancing the anticancer activities. We next confirmed that BGC2a consistently exhibited higher cytotoxicity than AF in pancreatic cancer cells (CAPAN-2 and PANC-1), melanoma (B16), and murine acute myeloid leukemia (BaF3-ITD). BGC2a’s full anti-cancer potential will be further explored in other malignant cancers, including breast and liver cancer.

Cancer cells rely on the cellular antioxidant defense system to avoid ROS-stimulated cell death [45]. The agents targeting those antioxidant systems to stimulate ROS generation have been proven effective in eliminating cancer cells. TrxR is an important antioxidant enzyme, and its inhibitors have demonstrated promising anti-cancer efficacy [46,47]. Although our best bi-gold compound BGC2a increased intracellular ROS, the increased ROS was not identified as the lethal cause in lung cancer cells. The pretreatment of the antioxidant agent VC failed to prevent the apoptotic effect induced by BGC2a, although the ROS level was significantly reversed. Arner and co-workers previously reported that the knockdown of TrxR1 by 90% using siRNA did not cause much cell death [22], validating our hypothesis that TrxR inhibition might not be the decisive factor of gold-based compounds.

**Figure 6:**
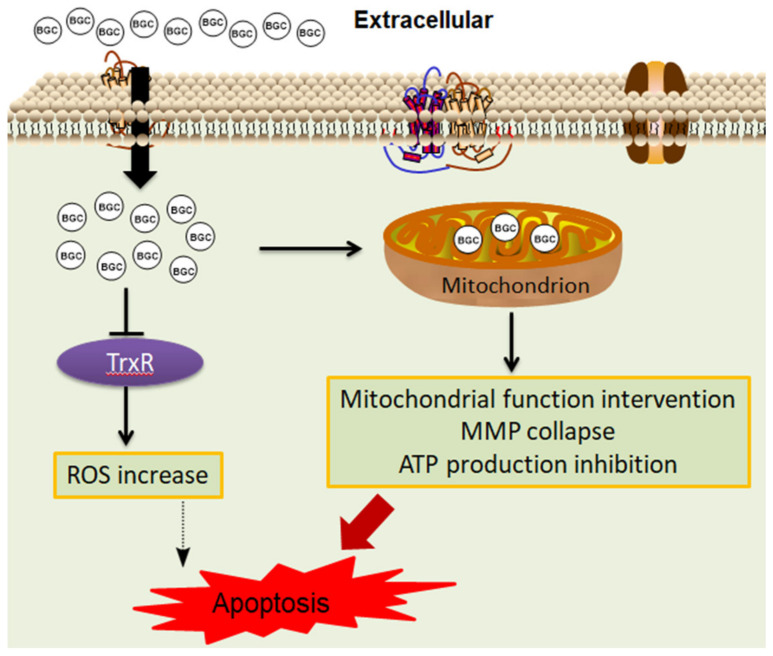
The schematic diagram of the action mechanism of BGC2a.

Our study further showed that compound BGC2a significantly increased its accumulation in the negatively charged mitochondria, inhibited the mitochondrial function, and down-regulated the protein levels of complex I and IV. While BGC2a increased both mitochondrial ROS and global ROS levels, its mitochondria-targeting ability seemed to play a central role in the anticancer activity of BGC2a, as summarized in Figure 6. It is worth noting that the content of BGC2a in the cytosol was higher than in mitochondria. However, a significant proportion of BGC2a accumulated in mitochondria might be enough to impact the intrinsically dysfunctional mitochondria. Meanwhile, non-mitochondrial actions of BGC2a cannot be excluded, requiring further study.

The anti-cancer effects of BGC2a were further confirmed in the colony formation assay in which the compound significantly suppressed the colony numbers of A549 cells that are widely reported to contain a substantial amount of stem-like SP cells [48]. In addition, BGC2a reduced the SP cells of A549 cells and suppressed the gene expression of CSCs biomarkers ABCG2, CD133, OCT4, and SOX2. Finally, this new compound substantially suppressed the tumor growth of A549 xenograft in mice without showing noticeable adverse effects, while AF only showed moderate inhibition of A549 tumor growth in our previous work [19]. The in vivo antitumor effect was further confirmed in a xenograft model of pancreatic cancer that is highly aggressive and often resistant to standard drug treatment.

While preliminary anti-cancer efficacies of BGC2a were validated, there are also some limitations in our current study. The exact targets of BGC2a in mitochondria remain to be determined in the future, although BGC2a unambiguously targeted mitochondria. It remains elusive if BGC2a can impact other functions of mitochondria, such as the synthesis of nucleotide precursors. BGC2a suppressed tumor growth in both A549 and PANC-1 xenograft mice; however, slight weight loss (without statistical differences) was observed during the treatment, requiring further safety evaluation. In addition, further immunohistochemical studies to test apoptosis and proliferation markers caspase 3 and ki67, as well as the safety of BGC2a, are needed. More structural modifications are also needed to improve the anticancer potency and selectivity of BGC2a.

## 4. Conclusions

Our current study has discovered that increasing mitochondria-targeting capacity enhances the anticancer activity of gold-based compounds, providing new insights into the underlying mechanisms that should be of significant value for the further development of gold-based anticancer drugs.

## 5. Materials and Methods

### 5.1. Chemistry

Unless stated otherwise, all commercially available chemicals were directly used in reagent grade, and all the solvents were in analytical grade. Column chromatography was performed on silica gel (200–300 mesh), and reactions were monitored by thin-layer chromatography on a glass plate coated with silica gel with the fluorescent indicator (GF254) using UV light and phosphomolybdic acid. The gold-containing compounds were synthesized following reported procedures [49]. ^1^H-NMR, ^13^C-NMR, and ^31^P-NMR spectra were recorded at ambient temperature on a 400 MHz spectrometer (AV-400 Bruker) in CDCl_3_. Chemical shifts are given in ppm (δ) referenced to CDCl_3_ with 7.26 for ^1^H and 77.16 for ^13^C. All the synthetic chemical procedures, characterizations of series 1–3 compounds, and MGC2a are included in the Supplementary Materials.

### 5.2. Cells and Cell Cultures

Human lung cancer cell lines A549, HCC827, NCI-H460, and NCI-H1650, pancreatic cancer cell lines PANC-1 and CAPAN-2, melanoma cell line B16, and mouse hematopoietic progenitor cell line BaF3 transfected with FLT3/ITD (BaF3/ITD) were purchased and authenticated by the American Type Culture Collection (ATCC, Rockville, MD, USA). Examination of mycoplasma contamination was performed before all experiments. Cells were cultured in RPMI 1640 medium, Mycoys5A or DMEM (Gibco-BRL, Paisley, UK) supplemented with 10% or 20% fetal bovine serum (FBS, Gibco-BRL, Paisley, UK). All cells were incubated in a humidified incubator at 37 °C supplemented with 5% CO_2_.

### 5.3. Cell Viability

Cells were seeded in 96-well plates at a density of 3000–5000/well and incubated overnight. Suspension cells were seeded at a density of 3–5×10^4^/well without further incubation. Then, drugs were added to each well and co-incubated for 72 h. Finally, to each well was added 3-(4,5-Dimethylthiazol-2-yl)-5-(3-carboxymethoxyphenyl)-2-(4-sulfophenyl)-2H-tetrazolium (MTS, 20 μL) and incubated for 3 h at 37 °C. The absorbance was measured under 490 nm by a microplate reader (BioTek SYNERGY, Winooski, VT, USA). Cell proliferation inhibition by a drug was evaluated in the values of IC_50_ (the concentration of a drug causing 50% inhibition of cell growth), calculated by GraphPad Prism 7 software (San Diego, CA, USA). Experiments were carried out in triplicate.

### 5.4. Cellular TrxR Activity

A549 cells were seeded in the 6-well plate at a density of 5×10^5^/well and incubated overnight. Then gold compounds (0.1, 0.3 μM) were added into each well and incubated for another 4 h. Thioredoxin Reductase Activity Colorimetric Assay Kit (Cat No. K763–100, Abcam, Cambridge, MA, USA) was used to examine the cellular enzyme activity following the manufacturer’s instruction. Experiments were carried out in triplicate.

### 5.5. ROS Levels and Mitochondrial Membrane Potential (MMP)

A549 cells were seeded in the 6-well plate at a density of 5 × 10^5^/well and incubated overnight, and then gold compounds (1, 3, and 5 μM) were added into each well. After treating for 2h or 6 h, cells were collected, washed with PBS, and stained with 6 μM CM-H_2_DCFDA (Invitrogen, Waltham, MA, USA) for 30 min for global ROS, MitoSOX™ Red (5 μM, 20 min) for mitochondrial ROS, and Rhodamine-123 (1 μM, 20 min) for MMP [50]. The cells were washed twice and re-suspended in PBS, followed by flow cytometry analysis using FACS Calibur flow cytometer (BD Biosciences, San Diego, CA, USA) or CytoFLEX (BECKMAN COULTER, USA). 2 mM Vitamin C (VC) pretreatment for 2 h was applied to study a reversal effect on the ROS level induced by BGC2a. All experiments were carried out in triplicate.

### 5.6. Apoptosis Assay

A549 cells were seeded in 6-well plates at a density of 3×10^5^/well and incubated overnight. Then, they were cultured with the indicated concentrations (2 and 3 μM) of gold compounds for 24 h. Cells were collected, washed with PBS, and stained with Annexin-V-FITC and PI (KeyGEN, Nanjing, China) for analysis of cell viability using CytoFLEX (BECKMAN COULTER, Indianapolis, IN, USA) [5]. VC pretreatment was applied to study a reversal effect on the apoptosis induced by BGC2a.

### 5.7. ATP Detection

The intracellular ATP was measured by the CellTiter-Glo Luminescent Cell Viability Assay (Promega, Madison, WI, USA) according to the manufacturer’s instructions. First, A549 cells were seeded in 96-well plates at a density of 1 × 10^4^/well and incubated overnight. Then, the cells were treated with indicated gold compounds (1, 3, 5, and 10 μM) for 6 and 12 h, respectively. After cell number normalization, 100 µL of cells were transferred to a 96-well plate and mixed with 100 µL of the ATP assay reagents. The mixture was incubated at room temperature for 10 min, and then the luminescence was detected by the microplate reader (BioTek SYNERGY, Winooski, VT, USA). Experiments were carried out in triplicate.

### 5.8. Protein Extraction and Western Blot Analysis

After being treated with BGC2a in different concentrations (0.1, 0.3, and 1 μM) for 24 h, A549 cells were washed with ice-cold PBS. The cells were lysed in a modified RIPA buffer (150 mM NaCl, 50 mM Tris, 0.1% SDS, 1% Triton X-100, 0.5% sodium deoxycholate, 1 mM EDTA) with a protease inhibitor cocktail and a phosphatase inhibitor cocktail (Roche, Indianapolis, IN, USA) for 15 min on ice. The cell debris was removed by centrifugation at 12,000× *g* for 15 min at 4 °C. The concentration of proteins was measured using the BCA protein assay (ThermoFisher, Rockford, IL, USA). Protein lysates were analyzed by standard SDS-PAGE and transferred to nitrocellulose membranes. Subsequently, the membranes were blotted with specific primary antibodies overnight at 4 °C and then incubated with appropriate horseradish peroxidase-conjugated secondary antibodies before the signals were revealed by the ECL detection system (Keygen Biotech. Co., Ltd., Nanjing, China). Total OXPHOS Human WB Antibody Cocktail (CAT. No. ab110411, Abcam, Cambridge, MA, USA) was applied to measure complex I–V, and β-actin was used as the internal control. The intensity of bands on western blots was quantified by Image J [50].

### 5.9. Colony Formation Assay

A549 cells in 2 mL RMPI 1640 medium with 10% FBS were seeded in a 6-well plate at a density of 400/well and incubated overnight. Then, the cells were treated with indicated concentrations (0.1, 0.3, and 0.6 μM) of gold compounds for 14 days, and photos of colonies were taken under an inverted microscope at 50× magnification. The medium was removed, and the cells were washed with PBS twice and incubated with 1 mL methanol for 15 min. Then, 1 mL crystal violet (Crystal Violet Staining Solution, Beyotime, Shanghai, China) was added to each well, incubated at room temperature for 30 min, and washed with running water. After drying out, the colony number was counted by gel imaging and analysis system (ProteinSimple, Alphalmager HP, Santa Clara, CA, USA) [50].

### 5.10. Cellular Oxygen Consumption Assay

Oxygen consumption rates (OCR) of A549 cells upon AF and BGC2a treatment (both at 1 and 3 μM) were measured by the Seahorse Bioscience Extracellular Flux Analyzer (XF24, Seahorse Bioscience, Billerica, MA, USA) following the manufacturer’s instruction. The XF Assay Media was supplemented with glucose (5.5 mM) and pyruvate (1 mM), and the pH was adjusted to 7.4. Oxygen consumption was recorded approximately every 8 min after gradual additions of a gold compound at the indicated concentration (1 and 3 μM), oligomycin (1 μM), FCCP (1 μM), and antimycin A/rotenone (0.5 μM). After adding a gold compound, basal respiration was measured once achieving a steady state. Coupled respiration was expressed as the decrease from basal respiration after adding oligomycin. Maximal respiration was taken as the highest measurement after adding FCCP [5]. Experiments were carried out in triplicate.

### 5.11. Detection of SP Cells and Non-SP Cells

A549 cells were collected, re-suspended in pre-warmed RPMI 1640 medium containing 2% FBS at a density of 1 × 10^6^ cells/mL, and incubated overnight. Then, the cells were treated with the indicated concentrations (0.3 and 1 μM) of drugs for 24 h. The cells were incubated with Hoechst 33,342 (5 μg/mL) in the presence or absence of an ABC transporter inhibitor verapamil for 90 min at 37 °C with intermittent shaking. The cells were washed with cold PBS, re-suspended in PBS, kept at 4 °C, and finally sorted by flow cytometry analysis using MoFlo XDP Cell Sorter (Beckman Coulter, Brea, CA, USA) [50,51].

### 5.12. Detection of the Total and Mitochondrial Gold Content by Mass Spectroscopy

A549 cells were seeded in 25 cm^2^ cell culture flasks at a density of 4 × 10^6^/well and incubated overnight in a humidified atmosphere (37 °C, 5% CO_2_). Then, gold compounds were added to make a final concentration at 3 μM and incubated for 2 h. Mitochondria were collected following the manufacturer’s instructions for the Qproteome Mitochondria Isolation kit (Cat No. 37612, Qiagen, Hilden, Germany). Briefly, cells were centrifuged at 500 g for 10 min at 4 °C and washed with 1 mL 0.9% saline solution. The cell pellet was re-suspended in 1 mL ice-cold Lysis Buffer and incubated for 10 min at 4 °C on a shaker. After centrifuging the lysate at 1000× *g* for 10 min at 4 °C, the pellet was re-suspended in 1.5 mL of ice-cold Disruption Buffer. After complete cell disruption using a Potter homogenizer, the lysate was centrifuged at 1000× *g* for 10 min at 4 °C. The supernatant was carefully transferred to a clean 1.5 mL tube and centrifuged at 6000× *g* for 10 min at 4 °C. The pellet containing mitochondria was collected after the supernatant was removed. Finally, the isolated mitochondria and cells were treated with fuming HNO_3_ at 60 °C for 1 h and then diluted with distilled water. The mitochondrial and total intracellular gold content was measured by inductively coupled plasma mass spectrometry (ICP-MS, iCAP Q, Thermo Fisher Scientific, Waltham, MA, USA) [52]. Experiments were carried out in triplicate. The gold content in the cytosol was calculated by subtracting mitochondrial gold content from the total intracellular gold content.

### 5.13. RT-qPCR Assay

Total RNA was extracted from A549 cells using Trizol (Invitrogen). cDNA was generated from an equal amount of total RNA (1 μg) using Prime Script RT reagent kit with DNA Eraser (Takara Biotechnology, Dalian, Liaoning, China) after treatment of BGC2a (0.3 and 0.6 μM) for 24 h. Then, RT-PCR was performed to measure the levels of ABCG2, OCT4, CD133, and SOX2, using SYBR Premix Ex Taq II kit (Tli RNase H Plus, Takara Biotechnology, Liaoning, China) and the CFX96 real-time system (Bio-Rad Laboratories, Hercules, CA, USA). The RT-PCR amplification reaction program consisted of one cycle of 95 °C/30 s and 40 cycles of 95 °C/5 s → 60 °C/30 s. In addition, the expression of β-actin was measured as the internal control for normalization. Experiments were carried out in triplicate. The forward and reverse primer sequences were used according to our previous research [50,51], as shown in Table S1.

### 5.14. In Vivo Evaluation of the Antitumor Effect

Animal experiments were conducted in compliance with a protocol approved by the Institutional Animals Care and Use Committee of Sun Yat-sen University Cancer Center and carried out in Center of Experiment Animal of Sun Yat-sen University (North Campus, approval number L102012016060L). Immune-deficient BALB/c nude mice (female, 5-week-old, 7 mice/group) were purchased from Vital River (Beijing, China) and cared for according to the guidelines of the Laboratory Animal Unit of Sun Yat-sen University. Mice were raised in specific pathogen-free environment in a ventilated caging system (7 mice at most per cage on small-particle corncob contact bedding). 4 × 10^6^ A549 cells were subcutaneously injected into the back flanks of BALB/c nude mice. After tumor volumes reached 30–50 mm^3^, the mice were randomly divided into three groups. Each group was treated with 3 mg/kg of BGC2a (6 mice) reconstituted in a designed soluble system (96% Saline, 2% DMSO, 2% Solutol HS-15), or 3 mg/kg of ADM (dissolved in saline), or only solvent (7 mice). The mice were treated with BGC2a intravenously (i.v.) 3 times weekly (every Monday, Wednesday, and Friday) and with ADM, i.p., twice weekly (every Monday and Friday). Mice weight and tumor sizes were measured 3 times a week by the formula: Volume  =  (Length  ×  Width^2^)/2. At the end of treatments, the mice were sacrificed via cervical dislocation following deep anesthesia. Tumors were collected, and their volume and weights were measured [51].

### 5.15. Statistical Analysis

Each experiment was repeated at least three times. The in vitro data were presented as mean ± S.D. Comparisons between groups were performed using Student’s unpaired t-test, one-way or two-way ANOVA, and Tukey’s post hoc test. A *p*-value of less than 0.05 is considered statistically significant.

## 6. Patents

Novel gold complexes and application thereof (2020). CN107043404B.

## Data Availability

The following are available online at http://www.researchdata.org.cn (accessed on 1 December 2021), and all requests for materials should be addressed to S. Wen and P Huang.

## Supplementary Materials

The following supporting information can be downloaded at: https://www.mdpi.com/article/10.3390/ijms232012169/s1.
