# Supplementary material for: Developing Bi-Gold Compound BGC2a to Target Mitochondria for the Elimination of Cancer Cells
Source: Int J Mol Sci. 2022 Oct 12;23(20):12169. doi: 10.3390/ijms232012169 (PMC9602679; doi:10.3390/ijms232012169)
Supplement: Supplementary file 1 [file ijms-23-12169-s001.zip › ijms-1941368-supplementary.pdf]

Supplementary Material

Content

Supplementary figures……………………………………………..… ……………S3

Table S1………………………………………………………………..…………S7

Experiments……………………………………………………………………..S8

In vivo evaluation of anti-tumor effect of BGC2a in PANC1 model………………S8

Chemical synthesis of gold compounds……..…………..…………..……………..S9

Reference………………………………………………………………………..S14

Supplementary figures

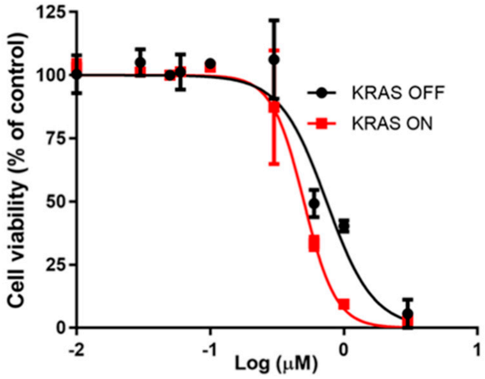

Figure S1. The inhibitory effects on K-RAS on/off HEK-293 cell lines by BGC2a.

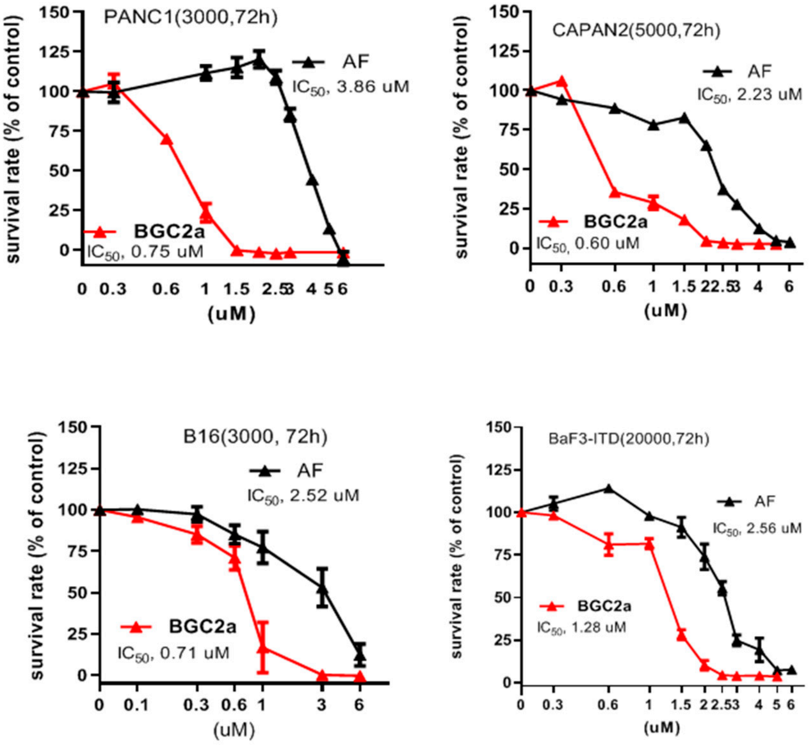

Figure S2. The anticancer activities of BGC2a and AF against pancreatic (PANC-1, CAPAN-2), melanoma (B16), and leukemia (BaF3-ITD) cancer cell lines.

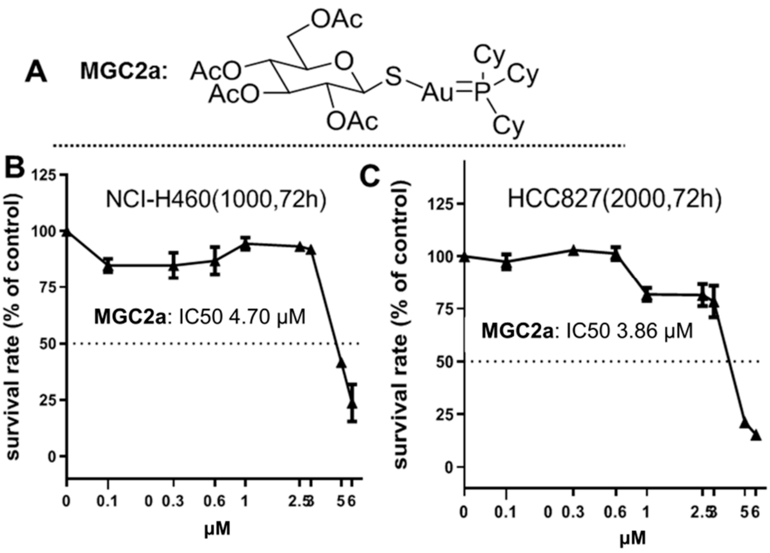

Figure S3. (**A**) AF derivative MGC2a carrying cyclohexyl substituent and (**B**) its anticancer activities in HCC827 and NCI-H460 cells.

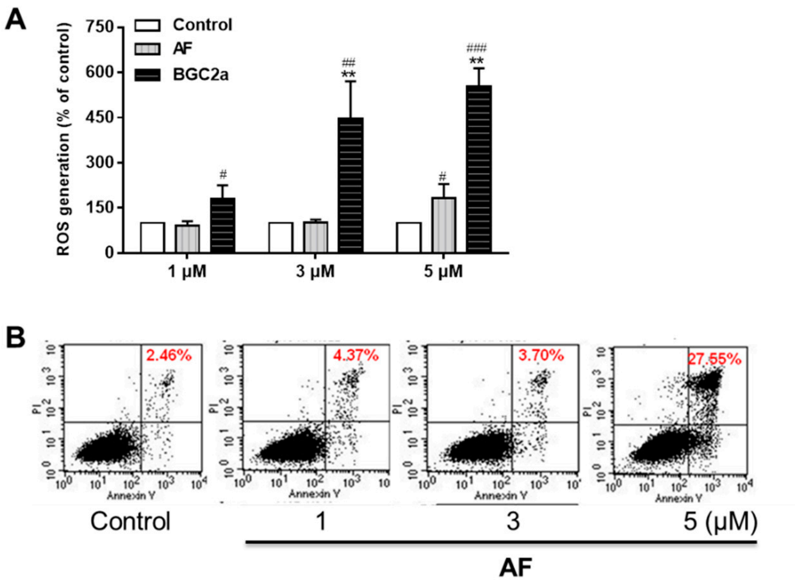

Figure S4. The impact of AF on cellular ROS and apoptosis. AF showed much weaker effects in inducing (**A**) ROS production and (**B**) apoptosis. (**) $P < 0.01$ *vs* the AF group; ($^{\#}$) $P < 0.05$, ($^{\#\#}$) $P < 0.01$, ($^{\#\#\#}$) $P < 0.001$ *vs* the Control group.

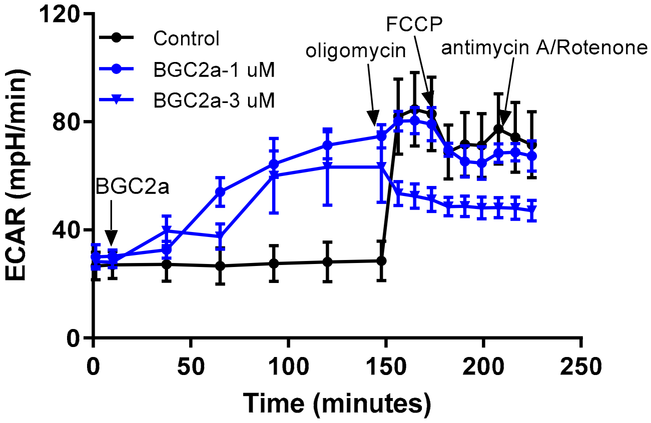

Figure S5. The calculated ECAR results upon BGC2a.

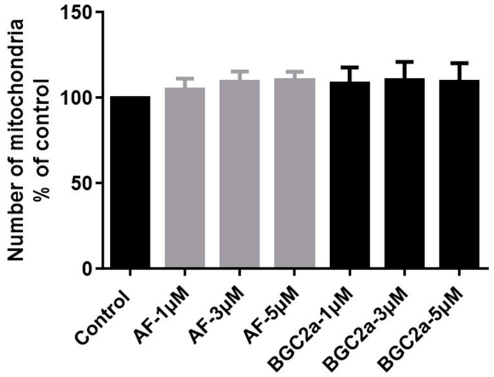

Figure S6. BGC2a and AF did not alter the mitochondria biogenesis.

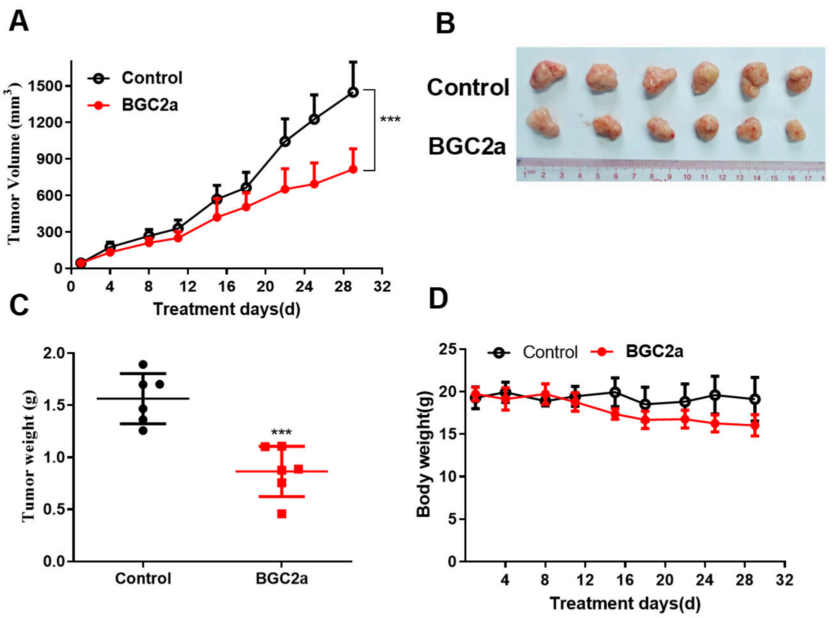

Figure S7. BGC2a suppressed the tumor growth in PANC-1 xenograft model. (**A)** The measured tumor volumes. Tumor volume was measured by electronic caliper and calculated using the formula volume (0.5 × length × width^2). (***) P < 0.001 *vs* the control group. (**B**) The tumor figure after sacrifice of mice. (**C**) Quantification of the tumor weights from **B**. (***) P < 0.001 *vs* the control group. (**D**) Body weights of the treated mice. Mouse weight was measured 3 times a week.

Table S1. The sequences of primers used for SYBR Green real-time PCR

Gene	Forward	Reverse
OCT4	CTGGGTTGATCCTCGGACCT	CCATCGGAGTTGCTCTCCA
ABCG2	CAGGTGGAGGCAAATCTTCGT	ACCCTGTTAATCCGTTCGTTTT
SOX2	GCCGAGTGGAAACTTTTGTCG	GGCAGCGTGTACTTATCCTTCT
CD133	AGTCGGAAACTGGCAGATAGC	GGTAGTGTTGTACTGGGCCAAT
Beta-actin	TTCTACAATGAGCTGCGTGTG	GGGGTGTTGAAGGTCTCAAA

Experiments

In vivo evaluation of anti-tumor effect of BGC2a in PANC-1 model

Animal experiment was conducted in compliance with a protocol approved by the Institutional Animals Care and Use Committee of Sun Yat-sen University Cancer Center and were carried out in Center of Experiment Animal of Sun Yat-sen University (North Campus). Immune-deficient BALB/c nude mice (female, 5-week-old, 7 mice/group) were purchased from Vital River (Beijing, China), and cared for according to the guidelines of the Laboratory Animal Unit of Sun Yat-sen University. Mice were raised in specific-pathogen free environment in a ventilated caging system (7 mice per cage on small-particle corncob contact bedding). 2×10^6 PANC1 cells were subcutaneously injected into the back flanks of BALB/c nude mice. After tumor volumes reached 30-50 mm^3, the mice were randomly divided into different groups. Each group was treated with 3 mg/kg of BGC2a reconstituted in a designed soluble system (96% Saline, 2% DMSO, 2% Solutol HS-15) and treated with 3 mg/kg of BGC2a or only solvent for the PANC-1 model. The mice were treated with BGC2a intraperitoneally (*i.p.*) for PANC-1 tumor model, respectively, 3 times weekly (every Monday, Wednesday, and Friday). Mice weight and tumor sizes were measured 3 times a week by the formula: Volume = (Length × Width^2)/2. In the end of treatments, the mice were sacrificed via cervical dislocation. Tumors were collected band their volume and weights were measured.

Chemical synthesis of gold compounds

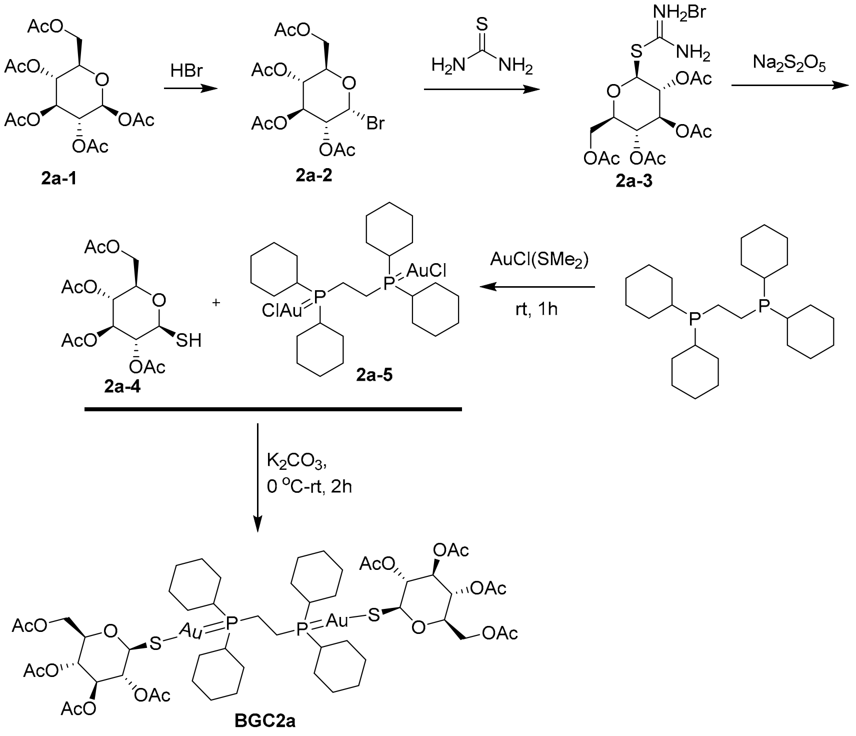

Synthesis of BGC2a: To a solution of 2a-1 (4.8 g, 12.30 mmol) in anhydrous CH_2Cl_2 (30 mL) cooled in an ice bath was added dropwise 33% HBr solution in AcOH (13 mL, 159.86 mmol). The mixture was allowed to be warmed to room temperature and stirred overnight. The mixture was diluted with CH_2Cl_2 (50 mL), washed with sat. aq. $NaCO_3$ (100 mL x 3) and brine (50 mL), dried over Na_2SO_4, and concentrated to get 2a-2. Without further purification, it was dissolved in acetone (50 mL), and thiourea (1.25 g, 16.42 mmol) was added. The mixture was heated to reflux for 5 h, concentrated under vacuo to get 2a-3, without further purification, it was dissolved in CH_2Cl_2/H_2O (60 mL, 2:1). Sodium metabisulfite (2.93 g, 15.93 mmol) was added, and the mixture was stirred at 55°C for 2 h. Then the mixture was extracted with CH_2Cl_2 (100 mL x 3) and the organic phases were dried over Na_2SO_4 and concentrated. The residue was purified by column chromatography on silica gel (EtAOc/PE=1/2) to give 2a-4 (4.5 g, 89%).

A solution of chloro(dimethyl sulfide)gold(I) (348 mg, 1.18 mmol) and 1,2-Bis(dicyclohexylphosphino)ethane (250 mg, 0.59 mmol) in CH_2Cl_2 (20 mL) was stirred at room temperature for 1 h to generate the intermediate 2a-5, then K_2CO_3 in minimum H_2O and

2a-4 (452 mg, 1.21 mmol) in CH_2Cl_2 (10 mL) was added. The mixture was stirred for 4 h at room temperature, the mixture was washed with H_2O (10 mL), then dried over Na_2SO_4, and concentrated. The residue was purified by column chromatography on silica gel (EtOAc/PE=1/1) to give BGC2a (also labelled as 2a) (540 mg, 59%). ^{1}H NMR (400 MHz, $CDCl_3$) δ 5.20 – 5.13 (m, 2H), 5.10 (d, *J* = 9.3 Hz, 2H), 5.00 (dt, *J* = 12.6, 9.6 Hz, 4H), 4.21 (dd, *J* = 12.2, 5.1 Hz, 2H), 4.06 (dd, *J* = 12.2, 2.4 Hz, 2H), 3.73 (ddd, *J* = 9.9, 5.0, 2.4 Hz, 2H), 2.06 (s, 6H), 2.05 (s, 6H), 2.04 (s, 6H), 2.07 – 2.01 (m, 4H), 2.00 (s, 6H), 1.96 (s, 6H), 1.92 (s, 12H), 1.74 (s, 4H),1.57 – 1.25 (m, 22H).^{13}C NMR (101 MHz, $CDCl_3$) δ 170.80, 170.26, 169.71, 169.57, 83.54, 77.89, 75.78, 74.32, 69.21, 63.19, 34.82, 34.67, 34.53, 30.17, 29.97, 29.19, 29.08, 26.83, 26.77, 26.70, 25.85, 21.26, 20.93, 20.77, 20.75. ^{31}P NMR (162 MHz, $CDCl_3$) δ 52.17.

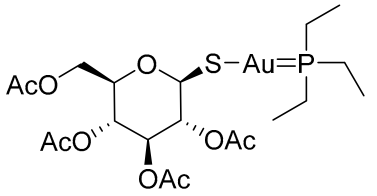

1a (Auranofin)

Compound 1a (Auranofin) was synthesized by a similar method as BGC2a. 1H NMR (400 MHz, CDC13) δ 5.16 (d, J = 9.5 Hz, 1H), 5.10 (dd, *J* = 11.8, 6.9 Hz, 2H), 5.01 – 4.92 (m, 1H), 4.24 (dd, *J* = 12.2, 4.8 Hz, 1H), 4.17 – 4.02 (m, 2H), 3.72 (s, 1H), 2.07 (s, 3H), 2.05 (s, 3H), 2.01 (s, 3H), 1.98 (s, 3H), 1.84 (dq, *J* = 15.4, 7.6 Hz, 6H), 1.23 (t, *J* = 7.7 Hz, 9H). The data agreed with the reported spectrum.[1]

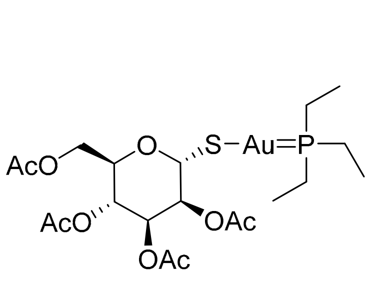

1b

Compound 1b was synthesized by a similar method as BGC2a. ^{1}H NMR (400 MHz, $CDCl_3$) δ

5.92 (dd, J = 9.9, 2.7 Hz, 1H), 5.77 (s, 1H), 5.47 – 5.34 (m, 1H), 5.25 (t, J = 10.0 Hz, 1H), 4.70 (d, J = 10.0 Hz, 1H), 4.30 (dd, J = 12.3, 3.7 Hz, 1H), 3.97 (d, J = 12.1 Hz, 1H), 2.08 (s, 3H), 2.04 (s, 3H), 1.96 (s, 3H), 1.90 (s, 3H), 1.85 – 1.70 (m, 6H), 1.13 (dt, J = 17.7, 7.6 Hz, 9H). ^{13}C NMR (101 MHz, $CDCl_3$) δ 171.19, 170.52, 170.07, 169.98, 80.69, 77.55, 77.23, 76.91, 76.05, 69.50, 68.24, 67.13, 62.89, 21.33, 21.06, 20.99, 18.25, 17.92, 9.11. ^{31}P NMR (162 MHz, $CDCl_3$) δ 37.22. MS (ESI) $[M+H]^+$ m/z 679.2.

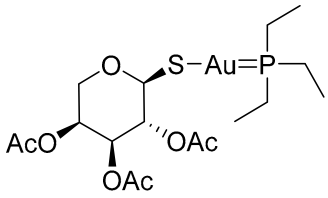

1c

Compound 1c was synthesized by a similar method as BGC2a. ^{1}H NMR (400 MHz, $CDCl_3$) δ 5.26 (d, J = 1.5 Hz, 1H), 5.21 (t, J = 9.5 Hz, 1H), 5.07 (d, J = 9.1 Hz, 1H), 4.96 (dd, J = 9.8, 3.6 Hz, 1H), 2.10 (s, 3H), 2.08 (s, 3H), 1.99 (s, 3H), 1.92 – 1.76 (m, 6H), 1.22 (dt, J = 18.4, 7.6 Hz, 10H). ^{13}C NMR (101 MHz, $CDCl_3$) δ 170.63, 170.50, 170.06, 84.09, 77.55, 77.23, 76.91, 75.03, 72.00, 69.42, 67.87, 21.47, 21.20, 20.99, 18.38, 18.05, 9.13. ^{31}P NMR (162 MHz, $CDCl_3$) δ 36.90. MS (ESI) $[M+H]^+$ m/z 607.2.

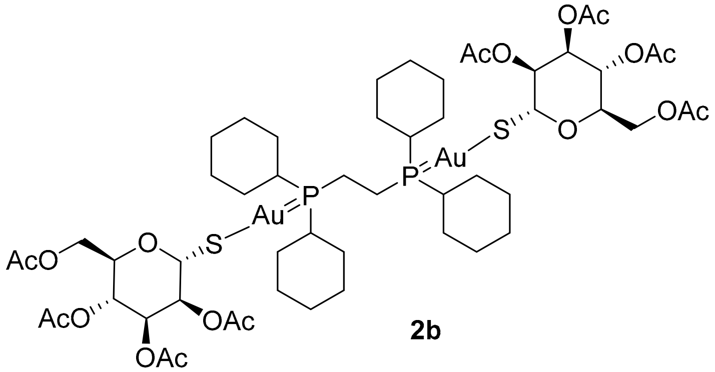

2b

Compound 2b was synthesized by a similar method as BGC2a. ^{1}H NMR (400 MHz, $CDCl_3$) δ 5.93 (d, J = 9.9 Hz, 2H), 5.77 (s, 2H), 5.43 (s, 2H), 5.31 (t, J = 9.9 Hz, 2H), 4.73 (d, J = 9.9 Hz, 2H), 4.34 (d, J = 12.2 Hz, 2H), 4.01 (d, J = 12.3 Hz, 2H), 2.20 – 1.73 (m, 51H), 1.40 (s, 8H), 1.34 – 1.16 (m, 12H). ^{13}C NMR (101 MHz, $CDCl_3$) δ 171.05, 170.29, 169.99, 169.86, 80.60,

77.55, 77.23, 76.91, 76.16, 69.62, 68.37, 67.15, 62.92, 35.05, 34.90, 34.76, 30.24, 30.07, 29.86, 29.41, 29.32, 26.91, 25.90, 25.81, 21.28, 21.00, 20.93, 19.36. ^{31}P NMR (162 MHz, $CDCl_3$) δ 51.63.

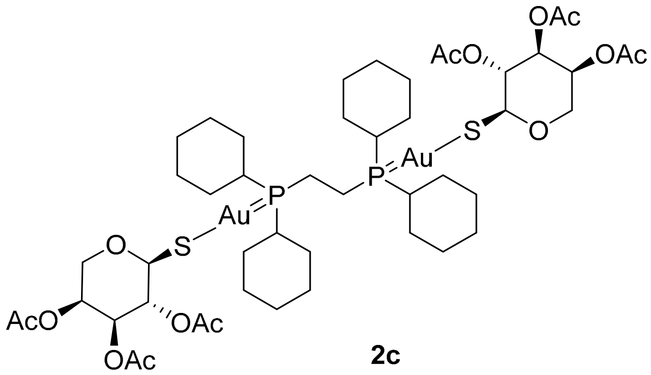

2c

Compound 2c was synthesized by a similar method as BGC2a. ^{1}H NMR (400 MHz, $CDCl_3$) δ 5.25 (d, *J* = 1.3 Hz, 2H), 5.17 (t, *J* = 9.2 Hz, 2H), 5.07 (d, *J* = 8.9 Hz, 2H), 4.98 (dd, *J* = 9.5, 3.6 Hz, 2H), 4.02 (dd, *J* = 13.1, 2.3 Hz, 2H), 3.68 (d, *J* = 13.0 Hz, 2H), 2.13 – 1.92 (m, 32H), 1.86 (d, *J* = 10.0 Hz, 12H), 1.73 (d, *J* = 10.2 Hz, 8H), 1.51 – 1.26 (m, 20H). ^{13}C NMR (101 MHz, $CDCl_3$) δ 170.59, 170.43, 169.77, 83.71, 77.55, 77.23, 76.91, 74.70, 71.89, 69.28, 67.56, 34.60, 30.07, 29.88, 29.27, 29.15, 26.84, 25.94, 21.41, 21.17, 20.96. ^{31}P NMR (162 MHz, $CDCl_3$) δ 51.87.

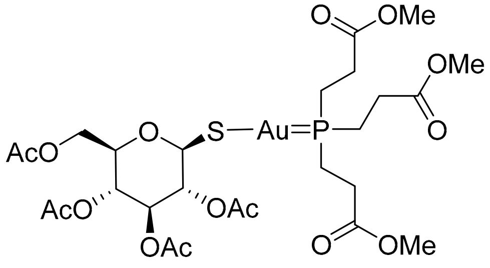

3a

Compound 3a was synthesized by a similar method as BGC2a. ^{1}H NMR (400 MHz, $CDCl_3$) δ 5.17 – 5.04 (m, 3H), 4.98 – 4.87 (m, 1H), 4.24 (dd, *J* = 12.3, 5.1 Hz, 1H), 4.10 (dd, *J* = 12.3, 2.2 Hz, 1H), 3.73 (s, 10H), 2.77 – 2.61 (m, 6H), 2.21 (dt, *J* = 15.8, 8.0 Hz, 6H), 2.06 (s, 3H), 2.06 (s, 3H), 2.00 (s, 3H), 1.97 (s, 3H). ^{13}C NMR (101 MHz, $CDCl_3$) δ 172.11, 171.97, 170.90, 170.34, 169.68, 83.50, 77.63, 76.13, 74.45, 69.17, 62.99, 52.46, 29.95,

29.91, 21.90, 21.57, 21.24, 20.91, 20.78. ^{31}P NMR (162 MHz, $CDCl_3$) δ 33.59. MS (ESI) $[M+H]^+$ m/z 853.2.

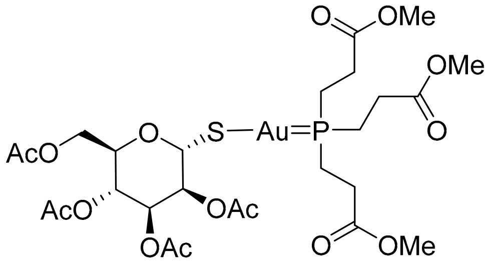

3b

Compound 3b was synthesized by a similar method as BGC2a. ^{1}H NMR (400 MHz, $CDCl_3$) δ 5.92 (dd, *J* = 10.0, 2.9 Hz, 1H), 5.77 (s, 1H), 5.47 – 5.39 (m, 1H), 5.29 (t, *J* = 10.0 Hz, 1H), 4.67 (d, *J* = 10.0 Hz, 1H), 4.33 (dd, *J* = 12.3, 4.0 Hz, 1H), 4.03 (d, *J* = 12.3 Hz, 1H), 3.71 (s, 9H), 2.66 (dt, *J* = 13.3, 7.9 Hz, 6H), 2.21 (dt, *J* = 15.8, 8.0 Hz, 6H), 2.11 (s, 3H), 2.08 (s, 3H), 2.02 (s, 3H), 1.94 (s, 3H). ^{13}C NMR (101 MHz, $CDCl_3$) δ 171.99, 171.85, 170.97, 170.33, 170.10, 169.93, 80.45, 75.89, 69.49, 68.51, 66.99, 62.84, 52.43, 29.87, 29.84, 21.84, 21.49, 21.15, 20.92, 20.86, 20.82. ^{31}P NMR (162 MHz, $CDCl_3$) δ 31.35. MS (ESI) $[M+H]^+$ m/z 853.2.

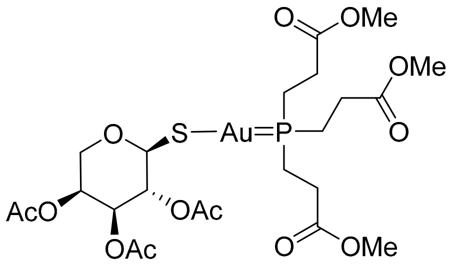

3c

Compound 3c was synthesized by a similar method as BGC2a. ^{1}H NMR (400 MHz, $CDCl_3$) δ 5.26 (s, 1H), 5.14 (d, *J* = 9.4 Hz, 1H), 5.05 (d, *J* = 9.1 Hz, 1H), 4.95 (dd, *J* = 9.6, 3.6 Hz, 1H), 4.03 (dd, *J* = 13.3, 2.1 Hz, 1H), 3.71 (s, 9H), 3.65 (s, 1H), 2.75 – 2.62 (m, 6H), 2.20 (dt, *J* = 10.0, 7.9 Hz, 6H), 2.12 (s, 3H), 2.06 (s, 3H), 1.97 (s, 3H). ^{13}C NMR (101 MHz, $CDCl_3$) δ 171.98, 171.83, 170.56, 170.24, 169.96, 83.88, 75.06, 71.76, 69.17, 67.79, 52.40, 29.85, 29.81, 21.85, 21.50, 21.26, 20.95, 20.79. ^{31}P NMR (162 MHz, $CDCl_3$) δ 31.51. MS

(ESI) $[M+H]^+$ m/z 781.2.

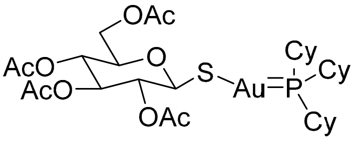

MGC2a

MGC2a: ^{1}H NMR (400 MHz, $CDCl_3$) δ 5.18 – 4.87 (m, 4H), 4.17 (dd, *J* = 12.1, 4.9 Hz, 1H), 4.02 (dd, *J* = 12.1, 2.2 Hz, 1H), 3.67 (ddd, *J* = 9.6, 4.7, 2.4 Hz, 1H), 2.02 (s, 3H), 2.00 (d, *J* = 6.5 Hz, 3H), 1.99 – 1.88 (m, 14H), 1.81 (s, 7H), 1.70 (s, 3H), 1.54 – 1.37 (m, 6H), 1.34 – 1.15 (m, 10H). ^{13}C NMR (101 MHz, $CDCl_3$) δ 171.00, 170.55, 169.82, 169.75, 83.53, 77.98, 75.78, 74.55, 69.34, 63.30, 33.74, 33.47, 30.98, 30.86, 27.36, 27.24, 26.12, 21.36, 21.03, 20.92, 20.88. ^{31}P NMR (162 MHz, $CDCl_3$) δ 56.76.

REFERENCES

1 Mirabelli, C. K., Johnson, R. K., Hill, D. T., Faucette, L. F., Girard, G. R., Kuo, G. Y. et al. Correlation of the in Vitro Cytotoxic and in Vivo Antitumor Activities of gold(I) Coordination Complexes. *J. Med Chem* **29**, 218-223 (1986).
